# Percutaneous Management of Right Ventricular Outflow Tract Obstruction in an Adult Patient After Atrial Switch: A Case for Targeted Infundibular Ablation

**DOI:** 10.1002/ccd.70328

**Published:** 2025-11-20

**Authors:** Vera Osberghaus, Kerstin Wustmann, Pinar Bambul Heck, Stanimir Georgiev, Andreas Eicken, Peter Ewert, Katarzyna Gendera

**Affiliations:** ^1^ Department of Congenital Heart Defects and Paediatric Cardiology, TUM University Hospital German Heart Center Technical University of Munich Munich Germany

## Abstract

Alcohol septal ablation is a well‐established treatment method for patients with hypertrophic cardiomyopathy. We present a case report of a patient with transposition of the great arteries (TGA) after a Mustard procedure, who developed significant right ventricular outflow tract obstruction (RVOTO), functionally resembling subaortic stenosis. Given the high surgical risk, interventional alcohol ablation of the conus branch of the right coronary artery (RCA) was successfully performed, resulting in a significant decrease in the pressure gradient.

## 1 Introduction

Surgical reinterventions in adult patients with congenital heart disease are often associated with increased procedural risks. This is particularly true for patients with a systemic right ventricle, who represent a challenging group as they reach the age of 30 or 40, when right ventricular function may begin to deteriorate and lead to clinical symptoms [1].

For this reason, repeated cardiac surgical procedures should be avoided in this patient population whenever possible.

On the one hand, successful surgical repair of subaortic stenosis does not always provide long‐lasting relief from obstruction, and restenosis may occur during follow‐up. On the other hand, for nearly three decades, alcohol septal ablation has served as a less invasive alternative to surgical myectomy in the treatment of symptomatic obstructive hypertrophic cardiomyopathy [2, 3].

## 2 Case Report

A 42‐year‐old woman (height 157 cm, weight 66 kg) with complete transposition of the great arteries (TGA), initially palliated with a central aortopulmonary shunt, repaired in childhood by atrial switch (Mustard procedure), and later reoperated for left ventricle–pulmonary artery (LV–PA) allograft implantation followed by revision of a stenotic pulmonary venous baffle 20 years ago, presented with heart failure symptoms, including bilateral lower‐limb edema, exertional dyspnea, and nocturia. She also reported intermittent episodes of resting tachycardia.

**Figure 1:**
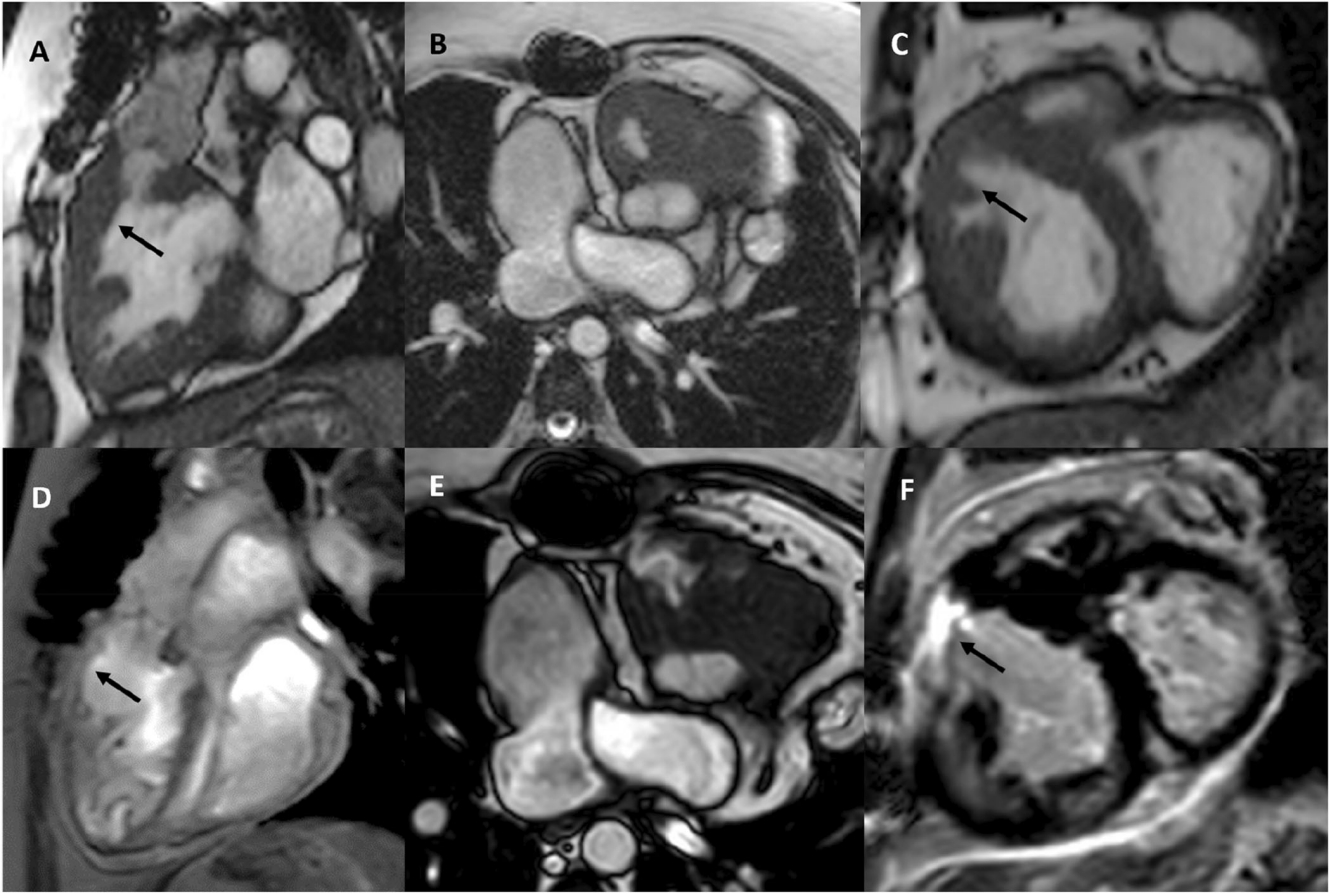
Cardiac magnetic resonance imaging demonstrating relevant right ventricular outflow tract obstruction before the procedure (A–C). Infundibular hypertrophy causing the obstruction is marked with an arrow. After the procedure, the infundibulum appears much thinner compared with the preprocedural images (D, arrow). Late gadolinium enhancement (LGE) depicts scar (arrow) tissue within the right ventricular outflow tract, consistent with the targeted ablation site (E, F).

Transthoracic echocardiography at presentation demonstrated subaortic stenosis with a peak Doppler velocity of 4.8 m/s and a corresponding maximal pressure gradient of 93 mmHg. The subaortic right ventricle (systemic ventricle) was markedly hypertrophied and showed mildly impaired systolic function. Tricuspid regurgitation was mild. Cardiac magnetic resonance imaging confirmed these findings (Figure 1A−C). Electrocardiography showed a sinus rhythm. Given the hemodynamic significance of the obstruction and the higher surgical risk, an interventional alcohol ablation of the right coronary artery (RCA) conus branch was planned.

The intervention was performed under sedation in a spontaneously breathing patient using a biplane fluoroscopy platform. Bilateral common femoral arterial access and left femoral venous access were obtained. A second arterial access was obtained to allow continuous monitoring of changes in the pressure gradient during the occlusion test. Intravenous unfractionated heparin (5000 IU) was administered. An invasive pressure gradient of 90 mmHg was recorded between the systemic right ventricle and the ascending aorta. The RCA was selectively cannulated and angiographically delineated.

**Figure 2:**
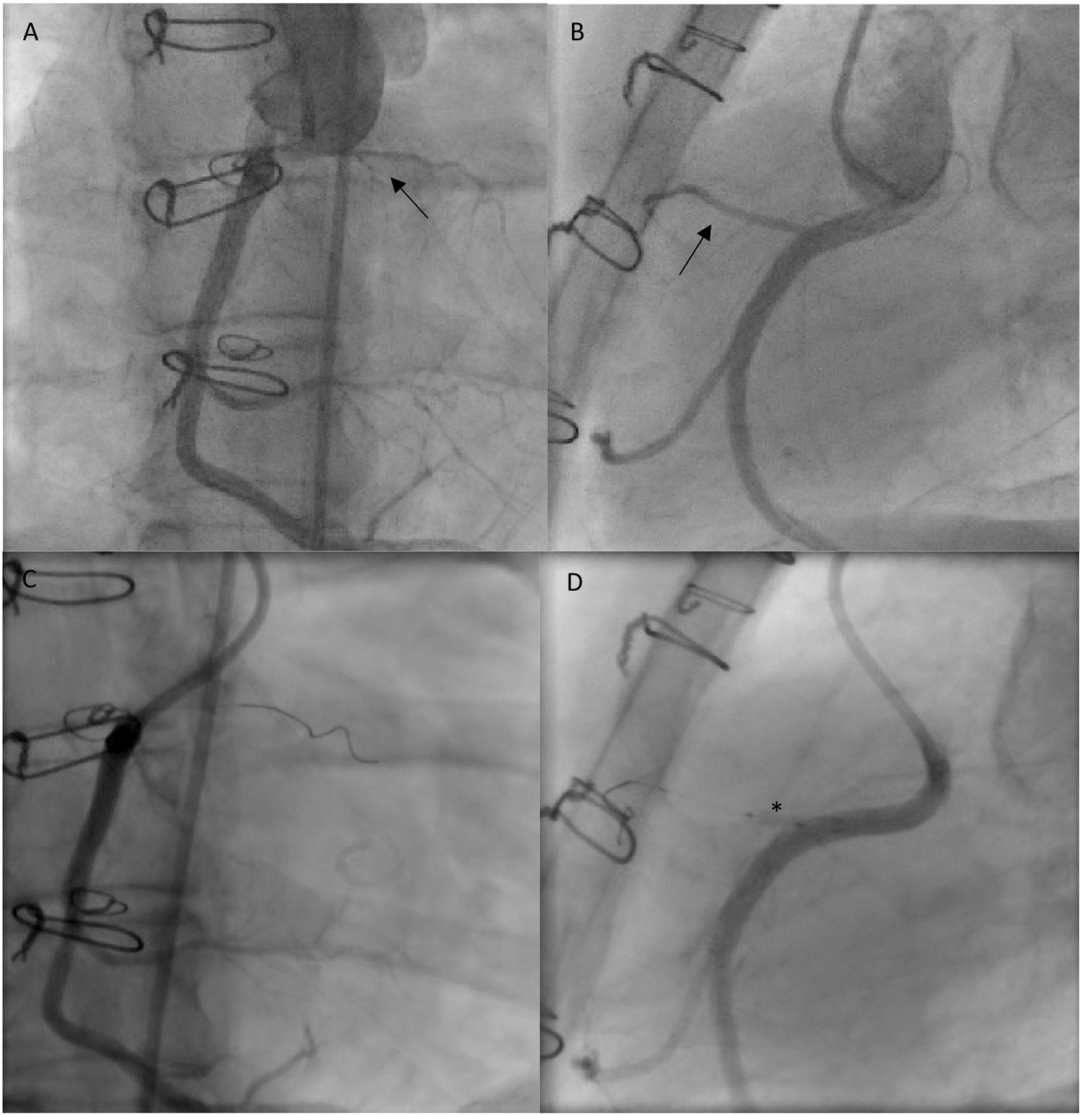
Right coronary angiography. (A, B) Before the ablation, the conus branch is indicated by an arrow. (C, D) Angiography demonstrating the absence of contrast agent flow into the conus branch during balloon occlusion for the ablation. The balloon is indicated by an asterisk.

The conus branch, situated directly above the hypertrophic infundibulum, was clearly identified (Figure 2A,B). No dedicated attempt was made to delineate the perfusion territory on echocardiography using intracoronary contrast. Angiographically, the embolized vessel appeared small and supplied only a limited myocardial region. The RCA was engaged with a 6 F Launcher coronary guiding catheter (Medtronic, Galway, Ireland), which provided optimal coaxial alignment with the posteriorly located right‐facing sinus in this patient with TGA. After selective engagement of the RCA, sequential test occlusions were performed with an Emerge over‐the‐wire balloon catheter (2.0 × 8 mm; Boston Scientific, Marlborough, MA, USA) at multiple levels, starting from the distal segment and progressing proximally, with the proximal occlusion resulting in a marked reduction in the transobstructive gradient (Figure 2C,D). A total of 1.8 mL of ethanol was injected into the proximal conus branch. Mild ST‐segment changes occurred during injection.

Post‐ablation, the invasive gradient decreased substantially to 25 mmHg. The procedure was completed without complications. The transient ST‐segment changes resolved completely during the subsequent 5‐day inpatient observation period. The peak troponin and CK levels were 2106 ng/L and 965 U/L, respectively, measured on the day after the procedure, and had declined by discharge. At 10 months postprocedure, the patient reported marked improvement in exercise tolerance; peripheral edema had resolved, and no anginal symptoms were present. Transthoracic echocardiography demonstrated a persistently low residual peak gradient in the RVOT, measuring up to 27 mmHg, corresponding to a peak Doppler velocity of 2.6 m/s. Follow‐up cardiac magnetic resonance imaging performed after 25.6 months (Figure 1D) demonstrated late gadolinium enhancement (LGE) indicating scar formation within the right ventricular outflow tract, consistent with the targeted ablation site (Figure 1E,F). The RV ejection fraction (RV‐EF) was 60%, comparable to the pre‐ablation value.

## 3 Discussion

We present a case report of a 44‐year‐old female patient with TGA after atrial switch according to Mustard, who developed significant RVOTO in the systemic position, resulting in subaortic stenosis.

The patient was successfully treated with alcohol infundibular ablation via a conus branch originating from the RCA.

The first catheter‐based septal ablation for obstructive hypertrophic cardiomyopathy was performed more than 30 years ago [2], initiating a therapeutic approach that has since benefited many patients suffering from symptomatic subaortic stenosis [3].

Although this method is widely used in patients with obstructive hypertrophic cardiomyopathy, there are, to date, only a few reports describing transcatheter alcohol ablation in patients with congenital heart disease. Das et al. reported on an 18‐year‐old patient with Tetralogy of Fallot who underwent complete percutaneous repair, including targeted alcohol ablation of the infundibulum, coil embolization of the conal artery, balloon angioplasty of the pulmonary valve, and closure of the ventricular septal defect (VSD) using an MFO Konar occluder [4]. The same group recently published a larger case series involving 35 patients with Tetralogy of Fallot treated with a combination of balloon pulmonary valvuloplasty and conal artery alcohol ablation to relieve RVOTO, along with VSD closure, and demonstrated promising intermediate‐term outcomes and minimal complications [5]. However, to date, our case report is the first to describe alcohol ablation of the conal artery for infundibular reduction in a patient with a systemic right ventricle and subaortic RVOTO.

The atrial switch procedure—developed independently by Senning in 1957 [6] and Mustard in 1963 [7]—was the standard surgical treatment for TGA until it was replaced by the arterial switch operation [8], widely adopted in the 1980s due to its more physiological restoration of ventricular‐arterial concordance. One of the key limitations of the atrial switch is the placement of the morphologic right ventricle in the subaortic, systemic position, where it is required to sustain systemic circulation over time.

Patients with a systemic right ventricle represent a particularly high‐risk population for open‐heart surgery and general anesthesia, especially as they reach the third or fourth decade of life, when right ventricular function declines or is impaired, leading to symptomatic heart failure or arrhythmias [1, 9]. Consequently, repeated open‐heart procedures should be avoided in this group whenever possible, and transcatheter solutions are increasingly being explored as less invasive alternatives. Given the elevated procedural risk and the presence of a systemic right ventricle, a transcatheter approach was selected to avoid the need for a more invasive surgical myectomy, which would have required a fifth median sternotomy in this patient.

After the procedure, the patient was observed for 5 days for close rhythm monitoring due to the potential risk of delayed arrhythmias (e.g., ventricular extrasystoles or tachycardia) following ablation. In addition, serial serum troponin levels were measured to assess myocardial injury. As this represented a novel therapeutic approach, extended monitoring was performed to ensure that the intervention did not result in any adverse effects.

Nevertheless, in patients with congenital heart disease—particularly those with TGA—coronary artery anomalies are common [10]. These may include anomalous origins, abnormal courses, or the presence of accessory branches, such as a dominant conal artery. For this reason, detailed coronary angiography is essential to assess the origin, course, and myocardial territory supplied by each coronary branch. A comprehensive understanding of the coronary anatomy and its relation to myocardial perfusion is critical before performing transcatheter alcohol ablation, to minimize the risk for myocardial ischemia, conduction disturbances, or inadvertent infarction of nontarget tissue.

In conclusion, alcohol ablation may be a feasible and effective treatment option even in selected cases with complex congenital heart disease. However, thorough preprocedural assessment of coronary anatomy and myocardial perfusion is essential to ensure procedural safety. Long‐term follow‐up is required to determine whether this approach affects the incidence of arrhythmias or the risk of restenosis in this specific patient population.

## Conflicts of Interest

The authors declare no conflicts of interest.
